# Identification of New, Functionally Relevant Mutations in the Coding Regions of the Human Fos and Jun Proto-Oncogenes in Rheumatoid Arthritis Synovial Tissue

**DOI:** 10.3390/life11010005

**Published:** 2020-12-23

**Authors:** René Huber, Sandra Augsten, Holger Kirsten, Roland Zell, Axel Stelzner, Hansjörg Thude, Thorsten Eidner, Bruno Stuhlmüller, Peter Ahnert, Raimund W. Kinne

**Affiliations:** 1 Experimental Rheumatology Unit, Department of Orthopedics, Jena University Hospital, Waldkliniken Eisenberg GmbH, 07607 Eisenberg, Germany; huber.rene@mh-hannover.de (R.H.); sandra.augsten@embl.de (S.A.); 2 Institute of Clinical Chemistry, Hannover Medical School, 30625 Hannover, Germany; 3 Structural and Computational Biology Unit, European Molecular Biology Laboratory, 69117 Heidelberg, Germany; 4 Institute for Medical Informatics, Statistics and Epidemiology, University of Leipzig, 04103 Leipzig, Germany; holger.kirsten@imise.uni-leipzig.de (H.K.); peter.ahnert@imise.uni-leipzig.de (P.A.); 5 Department for Cell Therapy, Fraunhofer Institute for Cell Therapy and Immunology, 04103 Leipzig, Germany; 6 Division of Experimental Virology, Institute for Medical Microbiology, Jena University Hospital, Friedrich Schiller University, 07747 Jena, Germany; roland.zell@med.uni-jena.de (R.Z.); axelstelzner@web.de (A.S.); 7 Institute of Transfusion Medicine, Jena University Hospital, 07747 Jena, Germany; h.thude@uke.de; 8 Department of Internal Medicine III, Division of Rheumatology & Osteology, Jena University Hospital, 07747 Jena, Germany; thorsten.eidner@med.uni-jena.de; 9 Division of Rheumatology and Clinical Immunology, Charité-Universitätsmedizin Berlin, Corporate Member of Berlin Institute of Health, Freie Universität and Humboldt-Universität, 10117 Berlin, Germany; bruno.stuhlmueller@charite.de; 10 Translational Centre for Regenerative Medicine, University of Leipzig, 04103 Leipzig, Germany

**Keywords:** rheumatoid arthritis, synovial membrane, fibroblast-like synoviocytes, transcription factor, AP-1, Jun, Fos, mutation, polymorphism

## Abstract

In rheumatoid arthritis (RA), the expression of many pro-destructive/pro-inflammatory proteins depends on the transcription factor AP-1. Therefore, our aim was to analyze the presence and functional relevance of mutations in the coding regions of the AP-1 subunits of the fos and jun family in peripheral blood (PB) and synovial membranes (SM) of RA and osteoarthritis patients (OA, disease control), as well as normal controls (NC). Using the non-isotopic RNAse cleavage assay, one known polymorphism (T252C: silent; rs1046117; present in RA, OA, and NC) and three novel germline mutations of the cfos gene were detected: (i) C361G/A367G: Gln121Glu/Ile123Val, denoted as “fos121/123”; present only in one OA sample; (ii) G374A: Arg125Lys, “fos125”; and (iii) C217A/G374A: Leu73Met/Arg125Lys, “fos73/125”, the latter two exclusively present in RA. In addition, three novel somatic cjun mutations (604–606ΔCAG: ΔGln202, “jun202”; C706T: Pro236Ser, “jun236”; G750A: silent) were found exclusively in the RA SM. Tansgenic expression of fos125 and fos73/125 mutants in NIH-3T3 cells induced an activation of reporter constructs containing either the MMP-1 (matrix metalloproteinase) promoter (3- and 4-fold, respectively) or a pentameric AP-1 site (approximately 5-fold). Combined expression of these two cfos mutants with cjun wildtype or mutants (jun202, jun236) further enhanced reporter expression of the pentameric AP-1 construct. Finally, genotyping for the novel functionally relevant germline mutations in 298 RA, 288 OA, and 484 NC samples revealed no association with RA. Thus, functional cfos/cjun mutants may contribute to local joint inflammation/destruction in selected patients with RA by altering the transactivation capacity of AP-1 complexes.

## 1. Introduction

Rheumatoid arthritis (RA), the most common rheumatic disease [1], is characterized by chronic inflammation and the destruction of cartilage and bone in multiple joints [2]. In RA, activated fibroblast-like synoviocytes (FLS) are a major component of the hyperproliferative, abnormally transformed, and invasive synovial membrane (SM) [3], the so-called pannus tissue [4]. FLS contribute to extracellular matrix turnover and joint destruction [5] by secretion of pro-inflammatory cytokines/chemokines and tissue-degrading enzymes [2,6] and the interaction with immune cells, e.g., macrophages, T-, or B-cells [6]. A variety of these effector molecules (such as interleukin (IL-) 1β, tumor necrosis factor, and matrix metalloproteinases (MMP)-1, -3, and -13) is regulated at the transcriptional level via the transcription factor activator protein (AP-) 1, classically a homo- or heterodimer of fos/jun proto-oncogene family members, including cfos, cjun, junB, and junD [7,8]. Amongst others, the transactivation activity of AP-1 is regulated by post-translational modifications of its subunits, e.g., via pro-inflammatory enzymes such as cJun N-terminal kinase (JNK) [9] and glycogen synthase kinase 3 [10]. Thus, AP-1 appears to be strongly involved in pro-inflammatory and pro-destructive processes [7] and represents one of the transcription factors contributing to the pathogenesis of RA [11]. The fos and jun family genes can be classified as “immediate-early response” genes, as they are rapidly induced by a variety of activating agents and show a very short half-life of only a few minutes [12]. Therefore, they can be regarded as markers of recent cell activation, e.g., in activated RA FLS [13], with clearly different biological activities of individual fos/jun family members [14]. In general, cFos and cJun are regarded as activating AP-1 subunits, whereas JunD and JunB mediate predominantly deactivating/inhibitory effects [15]. However, in specific situations, an activating capacity of JunB (e.g., in combination with Fos-related antigen 1 in collagen-induced arthritis [16]) and JunD (e.g., in macrophage activation during glumerulonephritis [17]) has been described.

It is well known that genetic alterations affecting functionally relevant domains of Jun and Fos proteins may critically influence AP-1 activity [18,19,20]. Recently, our group found several single nucleotide polymorphisms (SNPs) in the core promoters of cfos and cjun in RA and osteoarthritis (OA) patients modifying their transcriptional activation and we provided evidence for an association of the combined SNPs rs2239615 and rs7101 in the cfos promoter with the occurrence of knee OA [21]. For RA, associations with a variety of genetic variants have been identified in population genetic studies [22] and variants within the human leukocyte antigen gene complex appear to have the highest impact on RA development [22,23]. The presence of genetic variations in the fos and jun coding regions in the SM of RA patients (including somatic mutations) and their potential role for RA pathogenesis, however, have not been assessed yet. Therefore, the aim of this study was to analyze the incidence of genetic variations in the coding regions of cfos, cjun, junB, and junD in patients with RA, to investigate their functional relevance for AP-1 activity, and to assess potential associations between these SNPs/mutations and the occurrence of RA. To focus on the most relevant genetic alterations, a stepwise selection protocol was performed: (i) detection of polymorphic genetic variants in an initial screening population (including somatic mutations); (ii) identification of genetic variants with functional effects; and (iii) investigation of potential associations of the identified variants with RA in an extended cohort.

## 2. Results

### 2.1. Detection of Fos and Jun Single Nucleotide Polymorphisms (SNPs) and Mutations in Synovial Membrane (SM) Samples from Rheumatoid Arthritis (RA), Osteoarthritis (OA), and Normal Control (NC) Individuals

To identify genetic alterations with a potential functional relevance for the activity of AP-1 in RA, the occurrence of mutations and/or SNPs in the coding regions of fos and jun family members was initially assessed in (c)DNA samples from a limited number of affected patients. Therefore, peripheral blood (PB) and SM samples of RA (*n* = 11), OA (*n* = 10; disease controls), and NC patients/donors (*n* = 5; post-mortem samples from healthy individuals) were analyzed using the non-isotopic RNAse cleavage assay (NIRCA).

Within the cfos coding region, 4 genetic alterations were found (Table 1). In 7 RA, 3 OA, and 1 NC sample, a known polymorphism (T⟶C, rs1046117; [24]) was detected at position +252 (given in relation to the start codon), representing a silent SNP not affecting the respective amino acid (aa) Ser84. In addition, 3 novel mutations of cfos were identified, all located in the transactivation domain of the protein. A combined mutation (C361G/A367G) causing exchanges in codons fos121 and 123 (Gln121Glu/Ile123Val) was exclusively present in 1 OA patient. This mutant allele of cfos was not further investigated due to its unique occurrence in OA, since we considered OA as disease control in this study. The other two mutations were exclusively found in RA samples: G374A, causing the aa exchange Arg125Lys (4 RA patients, described as “fos125”) and the double mutant C217A/G374A, responsible for the Leu73Met exchange in combination with Arg125Lys (1 additional RA patient, “fos73/125”). Since all cfos SNPs and mutations were observed in both PB and SM of the corresponding patients, they have to be regarded as germline mutations.

The analysis of jun family genes revealed no SNPs or mutations in the coding regions of junB and junD. In the cjun coding region, however, 3 novel mutations were found affecting the glutamine- and proline-rich domain of the protein (exclusively in RA patients; Table 1). In one case, the deletion of a base triplet (604–606ΔCAG; 2 RA patients) resulted in the loss of a glutamine residue (ΔGlu202, “jun202”) in a cluster of 5 glutamines without affecting the reading frame. In 1 RA patient, a C706T mutation could be identified yielding a Pro236Ser exchange (“jun236”). Finally, the silent mutation G750A was detected in 3 RA patients. All cjun mutations were exclusively present in the SM but not in the PB, identifying them as somatic mutations. In this initial screening group, mutations causing exchanges in the cfos and cjun aa sequences were observed in different patients and did not occur in combination.

### 2.2. Functional Analyses

**Figure 1:**
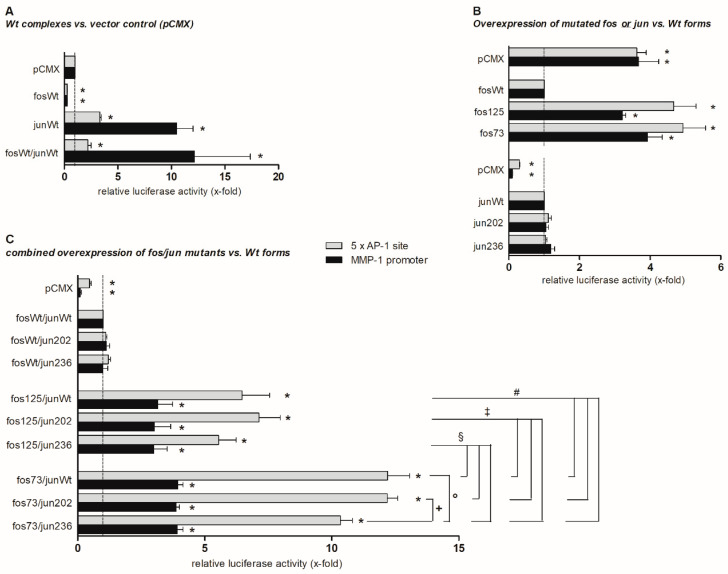
Effects of cjun and cfos mutations on reporter gene expression. The graph shows pentameric AP-1 site- and matrix metalloproteinase 1 (MMP-1) promoter-dependent expression of firefly luciferase 2 days following transfection of NIH-3T3 cells with wt (**A**) or mutated (**B**,**C**) cfos and cjun expression plasmids (determined in biological triplicates; means ± standard error of the mean). Firefly luciferase expression levels were normalized to renilla luciferase expression levels in the respective samples (transfection and normalization control). Results are presented as relative luciferase activity (in x-fold) related to the expression level in the presence of the control vector (**A**), cfos wt expression vector (**B**), or cfos and cjun wt vectors (**C**). For normalization and to correct for different transfection efficiencies, the renilla luciferase-expressing vector pRL-CMV was co-transfected. In all analyzed samples, luciferase expression was easy to detect and clearly exceeded background levels in non-transfected cells. * *p* ≤ 0.05 vs. controls, i.e., pCMX (**A**), fosWt (**B**), and fosWt/junWt (**C**); +, °, §, ‡, and # *p* ≤ 0.05 vs. respective other mutants; fos73 represents the double mutant fos73/fos125.

Considering the localization of the identified cfos and cjun mutations in functionally relevant protein domains (transactivation and glutamine-/proline-rich domain, respectively), their impact on protein function was assessed. Therefore, the effects of wildtype (wt) or mutated Fos and Jun proteins on the expression of firefly luciferase derived from reporter constructs containing either the human MMP-1 promoter or a pentameric AP-1 site were measured (Figure 1).

The cfos wt construct did not stimulate reporter gene expression when compared to the vector control (Figure 1A). In contrast, the cjun wt considerably increased the pentameric AP-1 site-dependent (3.5-fold) and, even more strongly, the MMP-1 promoter-dependent luciferase transcription (>10-fold). Co-expression of both cfos and cjun wt did not further increase cjun-mediated promoter construct activation (Figure 1A).

In comparison to the wt, the presence of the cfos mutations fos125 and fos73/125 (denoted as fos73 in Figure 1) led to a significant activation of AP-1 site-dependent (4.5- and 5-fold, respectively) or MMP-1 promoter-dependent (3- and 4-fold, respectively) gene expression. In contrast, cjun mutants jun202 and jun236 did not differ from the wt (Figure 1B). Moreover, the effects of a combined expression of cfos wt with these cjun mutants did not differ from those of co-expressed cjun/cfos wt (Figure 1C, upper part).

Overexpression of fos125 in combination with each of the cjun constructs (wt, jun202, jun236) led to a significantly stronger activation of the AP-1 site (6- to 7-fold; Figure 1C, central part) than the combined overexpression of cfos wt and cjun wt (Figure 1C, upper part) or fos125 alone (Figure 1B). In addition, combined overexpression of fos73/125 and cjun wt, jun202, or jun236 led to a further enhancement of the activation of the AP-1 site (up to 12-fold; *p* ≤ 0.05 for all respective comparisons; Figure 1C, lower part). When overexpressed together with fos125 or fos73/125, interestingly, jun236 showed a numerically or significantly smaller effect on the AP-1 site than cjun wt or jun202 (approximately 5% and 10% reduction, respectively, when compared to the wt). However, these differences were only observed with the “artificial” pentameric AP-1 site and not with the “physiological” MMP-1 promoter (Figure 1C, lower two parts).

### 2.3. DNA Binding Capacity of Expressed cFos and cJun Mutants

**Figure 2:**
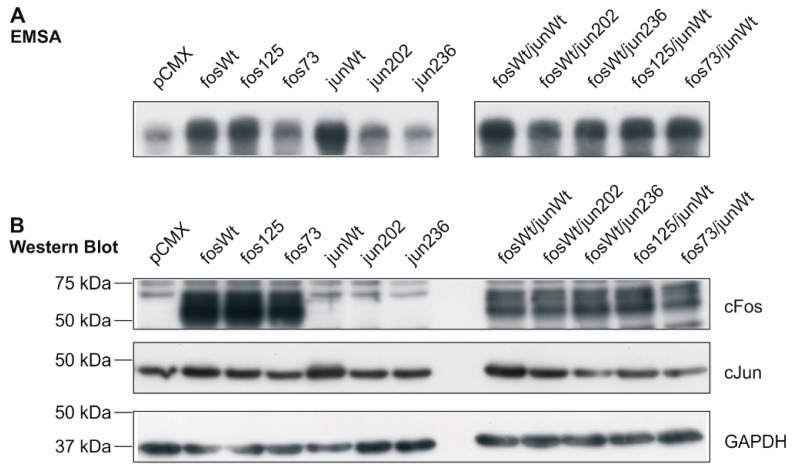
DNA binding activity of AP-1 complexes in transfected K4IM fibroblasts and protein expression of cfos and cjun variants. (**A**) Electrophoretic mobility shift assay (EMSA) of nuclear extracts from transfected K4IM cells. The phosphor-image exemplarily shows binding of radio-labelled AP-1 sites by AP-1 complexes formed in K4IM cells transfected with the indicated cfos and/or cjun variants (*n* = 1). (**B**) In the corresponding whole cell extracts, protein amounts of cFos and cJun following transfection were detected by Western Blot (*n* = 1). Loading control: glyceraldehyde 3-phosphate dehydrogenase (GAPDH); fos73: double mutant fos73/fos125.

The binding activity of AP-1 transcription factors consisting of the different cFos and cJun variants was determined by electrophoretic mobility shift assay (EMSA) in nuclear extracts of human K4IM fibroblasts transfected with expression vectors for the wt and mutants indicated in Figure 2. DNA activity of AP-1 was strongly increased in the presence of fosWt, fos125, and junWt when compared to the vector control pCMX. Variants fos73/125 and jun202 exhibited a less prominent, but still increased binding capacity. AP-1 complexes from jun236-containing samples, however, showed an unaltered binding activity comparable to vector control-transfected cells (Figure 2A, left). The combined expression of fosWt with junWt, jun202, and jun236 as well as fos125 and fos73/125 with the junWt unanimously resulted in a strong interaction of the respective AP-1 complexes with the ^32^P-labelled binding sites (Figure 2A, right). These data indicate that, following transfection, the expressed cFos and cJun proteins are present in the nucleus and able to associate with their DNA binding sites.

In parallel, the protein expression of cFos and cJun was analyzed in whole cell extracts using aliquots of the respective samples. As illustrated in Figure 2B, an overexpression of cfos wt, cfos mutants, and the cjun wt was detected in transfected K4IM cells. In the case of mutants jun202 and jun236, a clear overexpression was not detected. However, functional effects provoked by these mutants in NIH-3T3, RA, and OA FLS (see Section 2.2 and Section 2.4) indicated that in the functional assays, effective protein amounts were expressed.

### 2.4. Analysis of Matrix Metalloproteinase 1 (MMP-1) and Interleukin 6 (IL-6) Gene Expression in the Presence of cFos and cJjun Mutants

To assess the transactivation activity of the cfos and cjun variants in primary cells, vectors expressing the respective mutants were transfected (either alone or in combination) into primary FLS derived from RA or OA patients, in whom only the wt alleles could be detected. Subsequently, the mRNA expression of two AP-1-dependent genes, i.e., MMP-1 and Il-6, was measured by quantitative real-time polymerase chain reaction (qPCR).

**Figure 3:**
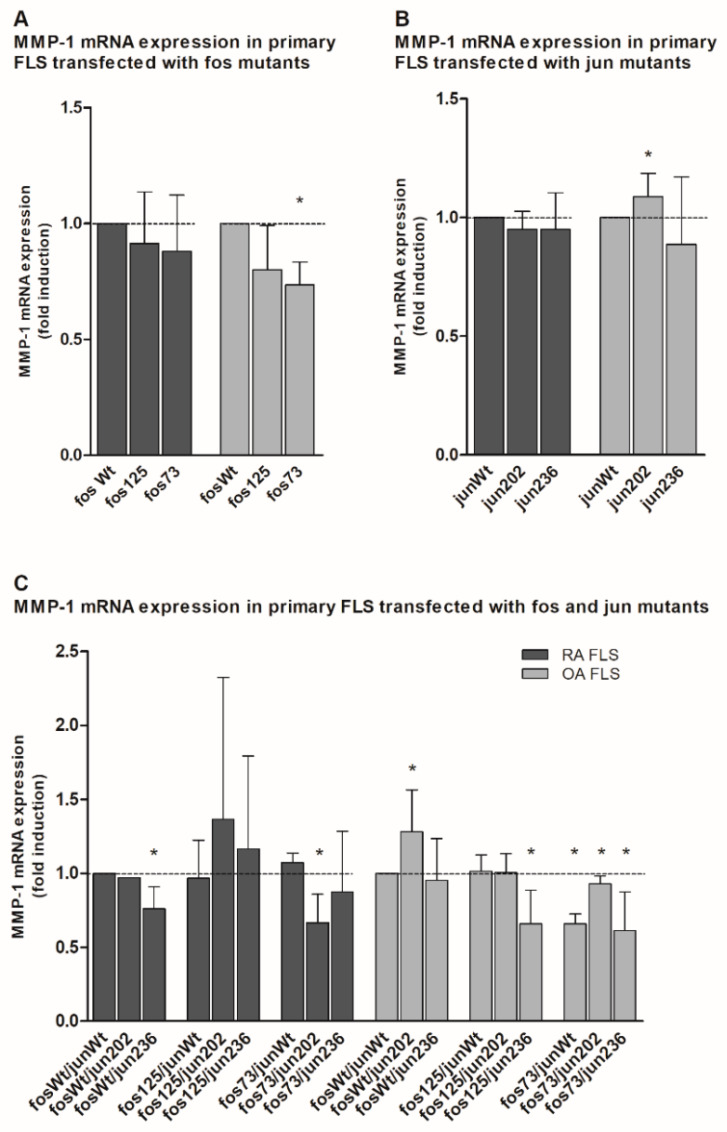
Quantitation of MMP-1 mRNA expression in jun-/fos-transfected primary human fibroblast-like synoviocytes (FLS). (**A**–**C**) The graph shows the MMP-1 mRNA expression (detected by qPCR) in primary FLS derived from RA and OA patients (without detectable genetic variations in the jun and fos genes; *n* = 3 each, mean ± SD) following transfection of cjun and/or cfos expression vectors. (**A**) MMP-1 expression in FLS transfected with the indicated cfos variants. (**B**) MMP-1 expression in FLS transfected with the indicated cjun variants. (**C**) MMP-1 expression in FLS transfected with combinations of cfos and cjun variants. MMP-1 mRNA levels in fosWt- (**A**), junWt- (**B**), or fosWt/junWt-transfected cells (**C**) were set as 1. Statistical analyses: Mann–Whitney U-test, * *p* ≤ 0.05 vs. the respective controls; fos73: double mutant fos73/fos125.

Interestingly, all cfos mutants and most of the cjun variants showed a tendency to repress MMP-1 mRNA expression in both RA and OA FLS when compared to the respective wt (Figure 3A,B). Only in the presence of jun202 was a significant induction of MMP-1 mRNA observed (Figure 3B).

This tendency was also seen when cfos and cjun mutants were expressed in combination. For instance, when compared to combined cfos and cjun wt, a significant downregulation was observed in RA FLS transfected with fosWt/jun236 or fos73/jun202 (with fos73 containing the double mutant fos73/125), as well as OA FLS transfected with fos125/jun236 and all combinations containing fos73/125 (Figure 3C). Again, only one approach including jun202 (i.e., fosWt/jun202) in OA FLS had a significantly positive effect on MMP-1 gene expression.

**Figure 4:**
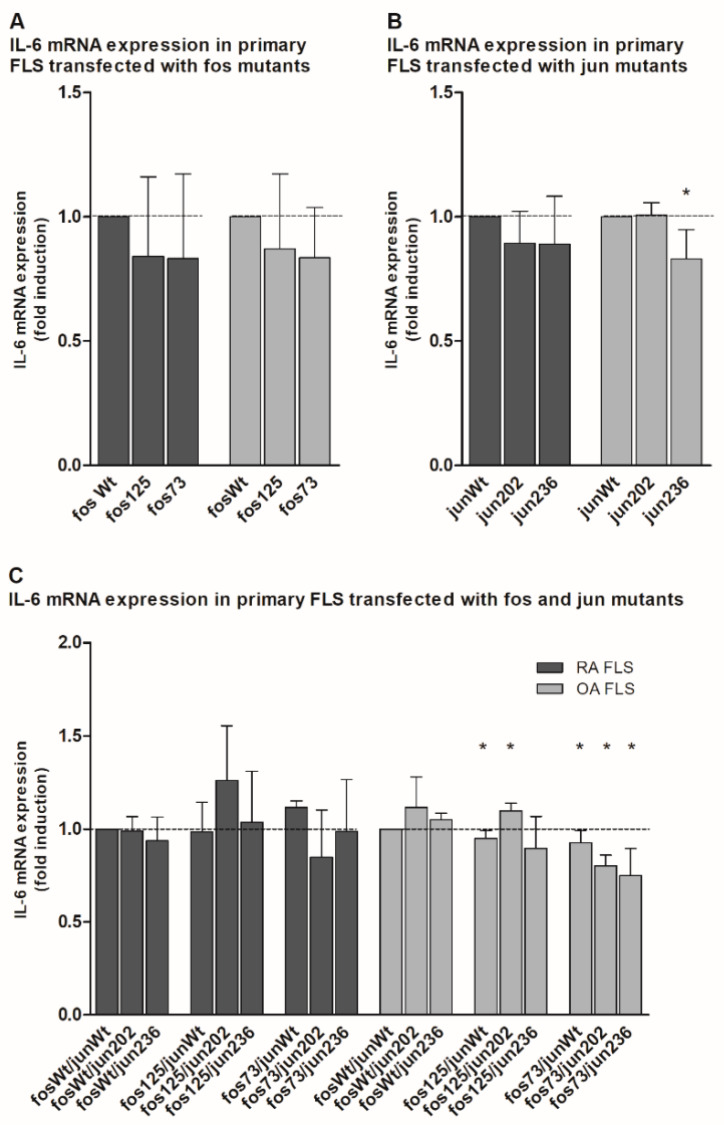
Quantitation of IL-6 mRNA expression in jun-/fos-transfected primary human FLS. (**A**–**C**) The graph shows the IL-6 mRNA expression (detected by qPCR) in primary FLS derived from RA and OA patients (without detectable genetic variations in the jun and fos genes; *n* = 3 each, mean ± SD) following transfection of cjun and/or cfos expression vectors. (**A**) IL-6 expression in FLS transfected with the indicated cfos variants. (**B**) IL-6 expression in FLS transfected with the indicated cjun variants. (**C**) IL-6 expression in FLS transfected with combinations of cfos and cjun variants. IL-6 mRNA levels in fosWt- (**A**), junWt- (**B**), or fosWt/junWt-transfected cells (**C**) were set as 1. Statistical analyses: Mann–Whitney U-test, * *p* ≤ 0.05 vs. the respective controls; fos73: double mutant fos73/fos125.

In the case of Il-6, comparable results were observed. Transfected mutants showed either no effect (jun202 in OA FLS; Figure 4B) or a numerical (and in the case of jun236 even significant) reduction (Figure 4A,B). Expressed together, only fos125/jun202-containing OA FLS induced a significant increase in IL-6 expression, whereas the fos125/junWt-expressing approach and all fos73/125-containing combinations in OA FLS showed significantly downregulated Il-6 mRNA levels (Figure 4C). Though less prominent (presumably due to the higher standard deviations), the mutants predominantly had very similar effects in RA FLS.

### 2.5. Allelic Distribution of Mutations

To analyze the allelic distribution of the identified mutations with potential functional effects on AP-1-dependent gene expression in RA (i.e., mutations detected in RA patients causing an aa exchange: fos73, fos125, jun202, and jun236), genotyping was performed in RA (*n* = 298), OA (*n* = 288), and NC (*n* = 484) whole blood DNA samples by single base extension and matrix assisted laser desorption ionization–time of flight (MALDI-TOF) multiplex analysis. Furthermore, we determined the frequencies of these mutations in the genome aggregation database gnomAD reporting on variants observed in a large number of sequenced human genomes and exomes [25,26].

In gnomAD, for cfos a non-synonymous amino acid exchange was also reported at amino acid position 73, however, a leucine to phenylalanine exchange was observed. In cjun, various deletions and duplications in the poly-glutamine cluster were reported, including the deletion of a single glutamine similar to our observed jun202 mutation (Table 2). In the analyzed patient/donor cohort (298 RA, 288 OA, and 484 NC samples), all identified functional germline mutations were genotyped as reference variant, i.e., none of the germline mutations was observed. Hence, no significant differences in the distribution of these mutations among different groups were detected.

## 3. Discussion

Progressive destruction of articular cartilage and bone, chronic inflammation, and the development of a transformed FLS phenotype in the SM are common features of RA [1,2] rendering the synovium its central pathophysiological component [3]. A variety of pro-inflammatory and pro-destructive proteins, such as chemokines/cytokines and matrix-degrading enzymes, decisively contribute to these processes [2]. On the transcriptional level, several of these genes are driven by the transcription factor AP-1, classically consisting of the subunits cFos, cJun, JunB, and JunD [7,8]. Thus, the presence and distribution of functionally relevant mutations and SNPs in the coding regions of these proto-oncogenes and their potential association with RA were analyzed.

Our initial analysis in blood and SM samples from RA and OA patients and post-mortem NC revealed that even in the small incipient screening population (11 RA, 10 OA, and 5 NC), a total of 6 mutations and 1 SNP could be detected (summarized in Table 1). The SNP rs1046117 represents a silent genetic alteration due to a synonymous T-to-C base exchange at the third position of codon fos84. Thus, no direct functional consequence on the aa sequence level results from the presence of this type of SNP [27] and it was not further studied. However, a concealed impact of such synonymous nucleotide exchanges on the gene function is still possible [27,28], e.g., due to biased codon usage [29] or modified translation efficiency and accuracy [30] during protein synthesis.

While the mainly deactivating AP-1 subunits JunB and JunD invariably showed the wt, equal numbers of mutations were observed in cfos and cjun, i.e., 3 mutations each. Interestingly, only the combined mutations affecting codons fos121 (C⟶G; Gln⟶Glu) and fos123 (A⟶G; Ile⟶Val) were selectively identified in 1 OA patient. The majority of genetic alterations (i.e., 5/6), however, were predominantly observed in RA samples, reflecting the frequent occurrence of mutations [31], rare variants [32], and other genetic alterations such as chromosomal aberrations [33,34] in RA. Among these, the G-to-A exchange at position 750 (given in relation to the first base of the start codon) caused a synonymous mutation in codon jun250. The loss of codon jun202 (ΔCAG) from a pentameric glutamine cluster in the proline- and glutamine-rich cJun domain also had no obvious functional consequences, since the transactivation capacity did not significantly differ from the wt. Although it is known that the domain harboring this cluster enables the interaction with the JNK [35], the loss of one glutamine residue had no perceptible impact on cJun activation in our experiments.

The remaining mutations unanimously led to aa exchanges with a potential impact on function and activity of the affected proteins [36], especially when considering the position of mutations in functionally relevant protein domains, i.e., the cFos transactivation domain and the glutamine-/proline-rich (interaction) domain in cJun. In cFos, mutations in codons fos73 (Leu⟶Met) and fos125 (Arg⟶Lys; in part occurring in combination) involve potentially significant changes in the protein structure. Though representing conservative exchanges between aliphatic/hydrophobic (fos73) and basic/polar aa (fos125), respectively, the mutations lead to altered size, charge distribution, and/or chemical reactivity of the respective residue [37]. A functional role for such aa exchanges has already been demonstrated in the mitochondrial NADH dehydrogenase subunit 2 (Leu⟶Met; [38]) and the signal transducer and activator of transcription 3 (Arg⟶Lys; [39]). Possible implications in protein structure and function are even more pronounced in the case of jun236, in which the hydrophobic, heterocyclic, “turned” proline is replaced by the polar, aliphatic, and more flexible aa serine [37]. This drastic (though localized) modification in protein structure may have significant consequences for protein chain conformation and resulting protein function such as protein–protein interactions. Thus, this mutation may contribute to the significantly less pronounced enhancement of fos125- and fos73/125-driven gene expression by jun236 (when compared to jun202 or junWt; Figure 1C).

Nonetheless, functional analyses revealed that the aa exchanges in cfos had a stronger influence on AP-1 transactivation activity than those in cjun (Figure 1). In comparison to the wt, which had no inducing effect on the luciferase reporter constructs (Figure 1A), a considerable induction of gene expression was observed in the presence of the mutants fos125 and fos73/fos125 (Figure 1B), indicating the susceptibility of AP-1-dependent genes to alterations in cFos. In contrast, overexpression of cJun wt proteins had a mildly activating effect on the artificial pentameric AP-1 promoter and caused a stronger activation of the physiological MMP-1 promoter (Figure 1A). This effect, however, was not modified by mutated variants jun202 or jun236, whose transactivating capacity was comparable to the wt (Figure 1B). The combined expression of cfos wt with either the wt or mutated versions of cjun also showed no differences in terms of reporter gene expression (Figure 1C). This suggests that under the present conditions, both wt and mutated cJun variants mainly mediate basal gene activation, as also observed in HeLa cells [40]. In contrast, mutants of cFos—which enhances stability, DNA binding activity [41], and transactivation activity [40] of AP-1 heterodimers—may be more powerful drivers of dysregulated AP-1 activity in RA.

Thus, the specific dimer composition of AP-1, which is regarded as one of the key factors for its impact on AP-1 target genes [42], may be of critical importance for dysregulated gene expression and its pathophysiological consequences in RA. Interestingly, activation of the pentameric AP-1 reporter by exogenous fos125 or fos73/125 was further increased upon co-expression with cjun wt or mutants, with a roughly comparable influence of the different jun variants (Figure 1C). This additive effect of cfos and cjun may be due to the inability of Fos family proteins to form stable homodimers—rendering Fos proteins dependent on the presence of suitable interaction partners [43]. It further highlights the necessity of balanced stoichiometric ratios among Fos and Jun proteins for the generation of active Fos-containing AP-1 complexes [44].

Interestingly, the functional consequences of the different cfos and cjun mutants were considerably different in immortalized NIH-3T3 fibroblasts and early-passage, primary OA or RA FLS. In the latter, jun202 (either alone or in combination with either fosWt and/or fos125) often had a stimulating effect on the MMP-1 and IL-6 mRNA expression, whereas in particular the co-expression of fos73/125 and the different cjun variants caused a significant downregulation of the mRNA expression of these pro-inflammatory/pro-destructive genes. These differences in cell lines and primary cells underscore the high complexity and (patho-) physiological variability of AP-1 effects in biological systems, which likely depend on: (i) the relative abundance of the different fos and jun components; (ii) the fine-tuned composition of the resulting AP-1 complexes; (iii) the functionally relevant, three-dimensional structure of these complexes; and (iv) their DNA binding and transactivation properties [7,14,42]. Whether crosslinks between the AP-1 pathway and other central signaling pathways [e.g., mitogen-activated protein kinases (MAPK; such as JNK, extracellular signal-regulated protein kinase (ERK), and p38), the phosphatidylinositide-3-kinase, and the Janus kinase/Signal transducer and activator of transcription (Jak/STAT) pathway [45,46,47]], known to be dysregulated in RA [46,48] and other diseases [49,50], contribute to the aforementioned differences, remains the focus of future studies.

In line with these aspects, aa modifying mutations in the jun/fos genes may affect additional features of AP-1. For instance, the interaction with activating kinases [35] or pro-inflammatory interaction partners, e.g., nuclear factor κB [51] or CCAAT/enhancer binding protein β [52], could be influenced. Further sensitive regions are sites acting as targets for protein-modifying enzymes mediating phosphorylation [42], SUMOylation [40], and others. Hence, proper post-translational modification in response to extra-cellular signals could be affected by mutations, with potential consequences for the ability of the cell to react towards environmental changes. Moreover, Jun and Fos protein stability and turnover could be affected (as described for a stabilizing point mutation affecting cJun-Ser243 [53]), i.e., effects potentially contributing to the discrepancy observed between jun/fos mRNA and protein expression in the SM from RA patients [54]. Furthermore, significantly altered stabilities of mutated Jun and Fos proteins might also have decisive consequences for their transactivation capacity, e.g., by prolonged gene expression cycles, altered interaction profiles with other transcription factors, or the establishment of unexpected feedback-loops. These aspects, however, have to be assessed in further studies.

Remarkably, in our study all detected mutations in cjun were somatic mutations that were absent in PB. It has been described before that in the RA SM somatic mutations are present in regulatory key genes such as p53 [55], affecting the functionality of the respective protein [56]. Interestingly, various different p53 mutations are found in SM tissue of individual patients. Moreover, number, type, and frequency of these mutations remarkably vary among different (sub-) areas within the SM (lining vs. sub-ling layer), a phenomenon that has been ascribed to the genotoxic stress present in the inflamed synovium [57]. In a kind of vicious cycle, such stressed and in part (semi-)transformed cells can enhance the extent of local inflammation [57], an effect possibly further supported by other mutations, for instance those affecting the inflammasome (which may also occur in mosaicism) [58]. Thus, it is reasonable to speculate that jun and fos family genes are also susceptible to the local development of de novo mutations due to inflammatory stress.

Despite the frequent occurrence of novel germline mutations in the initial screening population and the significant consequences of particular aa exchanges for protein function, no association of the identified alterations with (rheumatoid) arthritis was observed. To the best of our knowledge, this is consistent with the literature lacking reports on arthritides-associated jun/fos mutations (with the exception of an association of fos promoter variants with knee OA [21]), while a large variety of other susceptibility loci for RA is known [32]. This suggests that the variants observed are rare, distinct genetic alterations originating from individual or familial incidents. The low (or even absent) frequencies of these variants observed in a large collection of genetic variants (GnomAD, [25]) supports this assumption. These variants may be acquired as germline mutations during embryonic development, either de novo or ancestrally inherited [59], while somatic mutations can result from the local inflammatory, transformation-supporting milieu [60]. Type and frequency of arising mutations appear to depend on the specific nucleotide context and the surrounding region including aspects like existing histone modifications, GC content, and DNA methylation/hypersensitivity [61]. Despite the missing heritability in RA, it has been hypothesized that those causal variants (even though occurring in low frequencies) may account for an underlying genetic risk [32]. They may also contribute to the inter-individual and gene-specific variances in mRNA expression profiles observed in the RA SM [62].

In summary, the present data indicate that local mutations in the jun and fos family genes are qualified to modify the transactivation activity of the resulting AP-1 complexes and presumably contribute to particular pro-inflammatory/pro-destructive characteristics and disease progression in individual RA patients [63]. Our results also imply that genes strongly regulated by AP-1, e.g., those containing multiple copies of AP-1-recruiting tetradecanoylphorbol acetat- or cyclic adenosine monophosphate-responsive elements or AP-1-driven enhancers, may be more susceptible to the aforementioned fos and jun mutations than genes with more complex promoters containing a variety of binding sites for additional transcription factors, co-factors, or modification enzymes [43].

## 4. Materials and Methods

### 4.1. Patients, Tissue Samples, and Isolation of Primary Fibroblast-Like Synoviocytes (FLS)

Classification of patients/donors and collection of tissue samples was performed as described in [21]. FLS were isolated from OA and RA SM samples as published previously and used in early passage (up to passage 3; [64]). Whole blood samples were collected from RA (*n* = 298) and OA patients (*n* = 288) in the Departments of Orthopedics and Internal Medicine III/Division of Rheumatology and Osteology (Jena University Hospital) during routine blood withdrawal. Blood samples from NC donors (*n* = 484) were collected by the Institutes of Transfusion Medicine (University Hospitals Jena and Leipzig) during standard blood donation. The health status was controlled by hemogram, blood pressure measurements, individual medical history, and determination of virus parameters. If DNA was not immediately isolated, blood samples were stored at −20 °C. For NIRCA analyses of SM tissue samples (see Section 4.5), RA and OA samples were derived from a subpopulation of the RA/OA patients described above, while NC SM samples were derived from postmortem biopsies (Charité, University Medicine, Berlin, *n* = 5).

Informed consent was obtained from all patients and donors before blood sampling or joint replacement surgery. The experiments were carried out in accordance with the relevant guidelines and regulations. The study was approved by the Ethics Committees of the Friedrich Schiller University Jena (code: 0256-5/99, date: 15 May 1999 and code: 1154/-07/03, date: 30 June 2004) and the Humboldt University Berlin (code: EA1/193/10, date: 26 April 2012) in accordance with the Declaration of Helsinki. RA and OA patients were classified according to the respective criteria of the American College of Rheumatology [65,66] valid in the sample assessment period. Clinical characteristics of patients/donors are presented in Table S1. In all patients, the disease reached a level requiring joint replacement surgery (advanced joint destruction as assessed by radiography; categorized by the same team of clinicians), thus reflecting roughly comparable disease stages in the respective patient groups. Caucasian origin of the patients/donors was validated by self-disclosure and analysis of principal components of the genetic data.

### 4.2. DNA Preparation

Genomic DNA was prepared from 1070 whole blood samples. Extraction of DNA was performed using QIAamp DNA blood Mini kits or the Qiagen BioRobot EZ1 Workstation (Qiagen, Hilden, Germany) according to the manufacturer’s instructions. DNA concentration was determined using the Nanodrop ND-1000 system (PeqLab, Erlangen, Germany).

### 4.3. RNA Extraction, cDNA Synthesis, and Conventional Polymerase Chain Reaction (PCR)

RNA extraction, cDNA synthesis, and conventional PCR were performed as previously described [67,68]. Coding sequences of cjun, junB, junD, and cfos were amplified from genomic DNA (jun family genes) or cDNA (cfos, due to the presence of introns) using sequence-specific primers. Primer pairs contained binding sites for the restriction enzymes *Eco* RI and *Bam* HI or *Hind* III and *Not* I for subsequent cloning (see Section 4.4). Primer sequences and amplification protocols are presented in the Supplement (Table S2). Product specificity was confirmed by agarose gel electrophoresis and fluorescent cycle sequencing.

### 4.4. Cloning of DNA Fragments

For NIRCA analyses (see Section 4.5), DNA fragments of the wt cjun, junB, junD, and and cfos coding sequences were cloned into the vector pUC19 (Invitrogen, Darmstadt, Germany) by a standard ligation protocol using the restriction sites *Eco* RI and *Bam* HI (New England Biolabs, Frankfurt/Main, Germany).

For functional analyses (see Section 4.6), coding sequences of wt/mutated cfos and cjun were cloned into the expression vector pCMX [69] using the restriction sites *Hind* III and *Not* I. Vectors pUBT-luc-5AP1 (containing a pentameric AP-1 site (TGACTAA) cloned into the pUBT-luc vector [70]) and PXP1 (containing the MMP-1 promotor [71]) served as firefly luciferase reporter constructs for the activity of wt/mutated cJun and cFos.

### 4.5. Non-Isotopic RNase Cleavage Assay

For initial detection of SNPs and mutations in RA, OA, and post-mortem NC samples, the highly sensitive NIRCA was applied using the MutationScreener kit (Ambion, Austin, TX, USA). The assay was performed as previously described [72]. Subsequently, the existence of the observed base exchanges was validated and the precise position of each SNP or mutation was identified using Sanger sequencing [72].

### 4.6. Functional Analyses

Firefly luciferase reporter plasmids pUBT-luc-5AP1 or PXP1 were co-transfected with wt and/or mutated cfos and cjun expression vectors (500 ng each; see Section 4.4) into 1.5 × 10^5^ NIH-3T3 murine embryonic fibroblasts per well (cultured in Dulbecco’s Modified Eagle’s Medium + 10% fetal calf serum, 25 mM 4-(2-hydroxyethyl)-1-piperazineethanesulfonic acid, 100 U/mL penicillin, 100 mg/mL streptomycin, 2.5 mg/mL gentamicin; 6-well plates) using Polyfect (Qiagen) transfection reagent. The co-transfected renilla luciferase expressing vector pRL-CMV (10 ng/well) served as an internal transfection/normalization control [73]. Two days after transfection, cells were lysed with passive lysis buffer (Promega, Madison, WI, USA). Firefly luciferase expression was assessed via luminescence emission measurements using the dual luciferase assay system (Promega), normalized to renilla luciferase expression, and given as relative luciferase activity (i.e., firefly relative light units divided by renilla relative light units). In all cases, enzymatic activities of firefly and renilla luciferase were clearly detectable. Data of functional analyses were presented as means ± standard error of the mean (SEM). The Mann–Whitney U-test was applied to analyze differences among luciferase expression levels. Significant differences were accepted for *p* ≤ 0.05.

### 4.7. Quantitative Polymerase Chain Reaction (qPCR)

The qPCR was performed as previously described [54,67] using a LightCycler 2.0 (Roche Diagnostics, Mannheim, Germany) and primers specific for human MMP-1 (forward: 5′-gacctggaggaaatcttgc-3′, reverse: 5′-gttagcttactgtcacacgc-3′), IL-6 (forward: 5′-atgaactccttctccacaagcg-3′, reverse: 5′-ctcctttctcagggctgag-3′), and aldolase as a housekeeping gene (forward: 5′-tcatcctcttccatgagacactct-3′, reverse: 5′-attctgctggcagatactggcataa-3′) [74]. The general amplification protocol (25 cycles) was described in detail in [74] and involved initial denaturation (95 °C, 15 s), gene-specific settings for denaturation (95 °C, 10 s), primer annealing (MMP-1: 58 °C, 15 s; IL-6: 62 °C, 10 s; aldolase: 58 °C, 20 s), and amplification (68 °C, 20 s) as well as an additional heating step to melt potential primer dimers (81 °C, 8 s). Finally, a melting curve protocol (1 cycle) was applied. The fluorescence emitted by double-stranded DNA-bound SYBR-Green was measured once at the end of each additional heating step and continuously during the melting curve program. Concentrations of MMP-1 and IL-6 cDNA present in each sample were calculated by the LightCycler software using external standard curves (generated using 10-fold dilutions of plasmids containing the respective target sequence) and normalized to the aldolase content.

### 4.8. Preparation of Nuclear and Whole Cell Extracts

Nuclear extracts for the EMSA (see Section 4.9) and whole cell extracts for Western Blot analyses (see Section 4.10) were prepared from K4IM human fibroblast cells [75] transfected with expression plasmids coding for cfos and cjun wt and/or mutants (either alone or in combination) as previously described [76].

### 4.9. Electrophoretic Mobility Shift Assay

A detailed description of the EMSA is included in [76]. In brief, nuclear extracts (10 μg total protein) from K4IM cells were incubated with 2 × 10^8^ counts per minute of a ^32^P-labeled, double-stranded AP-1 oligonucleotide probe (sense strand only: 5′-CGC TTG ATG AGT CAG CCG GAA-3′; Promega) in 25 μL binding buffer (1 M Tris, 1 M boric acid, 0.02 M EDTA, 5% glycerol) supplemented with poly [dI-dC] (0.16 mg/mL) and 2 mM dithiothreitol. The samples were separated on a 4% polyacrylamide gel and scanned in a phosphor imager (BAS-1000, Fuji Photo Film, Tokio, Japan).

### 4.10. Antibodies, Sodium Dodecyl Sulfate Polyacrylamide Gel Electrophoresis (SDS-PAGE), and Western Blot

Sodium dodecyl sulfate polyacrylamide gel electrophoresis (SDS-PAGE) and Western Blot analysis were performed using standard protocols as described in [77]. The protein content in whole-cell extracts from K4IM cells was determined using the bicinchoninic acid assay (Pierce, Rockford, IL, USA). For cFos and cJun protein detection, SDS-PAGE (10%) was performed using 10 μL of each whole cell extract sample, followed by Western Blot analysis using the respective cFos and cJun antibodies from the TransAM AP-1 Family Kit (Active Motif, Rixensart, Belgium). The GAPDH antibody was purchased from Sigma-Aldrich (St. Louis, MO, USA) and the horseradish peroxidase conjugated secondary antibody from Santa Cruz Biotechnology (Santa Cruz, CA, USA). Proteins were visualized by chemiluminescence (Supersignal West chemiluminescent substrate; Pierce) using a Bio-Rad ChemiDoc apparatus [74].

### 4.11. Genotyping and Statistical Analysis

Genotyping was performed in RA (*n* = 298), OA (*n* = 288), and NC (*n* = 484) whole blood DNA samples by single base extension and MALDI-TOF multiplex analysis applying the GenoLink system (Bruker Daltonics, Bremen, Germany). Primers are shown in the Supplementary Materials (Table S3). For the design of genotyping primers, the software Calcdalton was used [78]. Reaction conditions were essentially the same as those described before [21,79].

## Figures and Tables

**Table 1:** Cfos and cjun mutations detected in synovial tissue of rheumatoid arthritis (RA) and osteoarthritis (OA) patients.

Mutations				Incidence of Mutations		
Gene	Description	nt Exchange	aa Exchange	RA	OA	NC
cfos	fos84	T252C *	none	7/11	3/10	1/5
	fos121/123	C361G A367G	Gln121Glu Ile123Val	0	1/10	0
	fos125	G374A	Arg125Lys	4/11	0	0
	fos73/125	C217A G374A	Leu73Met Arg125Lys	1/11	0	0
cjun	jun202	604–606ΔCAG	202ΔGln	2/10	0	0
	jun236	C706T	Pro236Ser	1/10	0	0
	jun250	G750A	none	3/10	0	0

* SNP rs1046117 (nt, nucleotide; aa, amino acid).

**Table 2:** Frequency analysis of mutations in GnomAD.

Observed Mutations			GnomAD [25,26] at aa Site	
**Gene**	Description	Position hg19 (aa Exchange)	ID	Frequency (n-Minor Alleles)
cfos	fos73/	chr14:75746655 (Leu73Met)	14-75746684-C-T	L73F: 3 × 10^−5^ (1)
	fos125	chr14:75746812 (Arg125Lys)	n.d.	n.d.
cjun	jun202	chr1:59248125-59248133 (202ΔGln)	1-59248123-GGCT-G	Q206del: 9 × 10^−4^ (204)
			1-59248123-G-GGCT	Q206dup: 2 × 10^−4^ (39)
			1-59248123-G-GGCTGCT	Q205_206dup: 2 × 10^−5^ (5)
			1-59248123-GGCT-G	Q205_206del: 5 × 10^−6^ (1)
	jun236	chr1:59248037 (Pro236Ser)	n.d.	n.d.

gnomAD, genome aggregation database v2.1.1; hg19, reference genome hg19; dup, duplication; del, deletion; n.d., not detected.

## Data Availability

The data presented in this study are available on request from the corresponding author.

## Supplementary Materials

Supplementary Materials can be found at https://www.mdpi.com/2075-1729/11/1/5/s1.

## Abbreviations

aa	amino acid
AP	activator protein
del,	deletion
dup	duplication
EMSA	electrophoretic mobility shift assay
FLS	fibroblast-like synoviocytes
GAPDH	glyceraldehyde 3-phosphate dehydrogenase
gnomAD	genome aggregation database
IL	interleukin
Jak	Janus kinase
JNK	cJun N-terminal kinase
MALDI-TOF	matrix assisted laser desorption ionization - time of flight
MMP	matrix metalloproteinase
NC	normal control
n.d.	not detected
NIRCA	Non-isotopic RNAse Cleavage Assay
OA	osteoarthritis
qPCR	quantitative real time polymerase chain reaction
RA	rheumatoid arthritis
SDS-PAGE	sodium dodecyl sulfate polyacrylamide gel electrophoresis
SM	synovial membrane
SNP	single nucleotide polymorphism
STAT	signal transducer and activator of transcription
wt	Wildtype

## References

[1] Firestein G.S., McInnes I.B. (2017). Immunopathogenesis of Rheumatoid Arthritis. Immunity.

[2] Bottini N., Firestein G.S. (2013). Duality of fibroblast-like synoviocytes in RA: Passive responders and imprinted aggressors. Nat. Rev. Rheumatol..

[3] Pap T., Dankbar B., Wehmeyer C., Korb-Pap A., Sherwood J. (2020). Synovial fibroblasts and articular tissue remodelling: Role and mechanisms. Semin. Cell Dev. Biol..

[4] Fearon U., Canavan M., Biniecka M., Veale D.J. (2016). Hypoxia, mitochondrial dysfunction and synovial invasiveness in rheumatoid arthritis. Nat. Rev. Rheumatol..

[5] Wollbold J., Huber R., Pohlers D., Koczan D., Guthke R., Kinne R.W., Gausmann U. (2009). Adapted Boolean network models for extracellular matrix formation. BMC Syst. Biol..

[6] Yoshitomi H. (2019). Regulation of Immune Responses and Chronic Inflammation by Fibroblast-Like Synoviocytes. Front. Immunol..

[7] Shiozawa S., Tsumiyama K. (2009). Pathogenesis of rheumatoid arthritis and c-Fos/AP-1. Cell Cycle.

[8] Wagner E.F. (2009). Bone development and inflammatory disease is regulated by AP-1 (Fos/Jun). Ann. Rheum. Dis..

[9] Yung J.H.M., Giacca A. (2020). Role of c-Jun N-terminal Kinase (JNK) in Obesity and Type 2 Diabetes. Cells.

[10] Hoffmeister L., Diekmann M., Brand K., Huber R. (2020). GSK3: A Kinase Balancing Promotion and Resolution of Inflammation. Cells.

[11] Okamoto H., Cujec T.P., Yamanaka H., Kamatani N. (2008). Molecular aspects of rheumatoid arthritis: Role of transcription factors. FEBS J..

[12] Shaulian E., Karin M. (2001). AP-1 in cell proliferation and survival. Oncogene.

[13] Kinne R.W., Boehm S., Iftner T., Aigner T., Vornehm S., Weseloh G., Bravo R., Emmrich F., Kroczek R.A. (1995). Synovial Fibroblast-Like Cells Strongly Express Jun-B and C-Fos Proto-Oncogenes in Rheumatoid- and Osteoarthritis. Scand. J. Rheumatol..

[14] Zenz R., Eferl R., Scheinecker C., Redlich K., Smolen J., Schonthaler H.B., Kenner L., Tschachler E., Wagner E.F. (2007). Activator protein 1 (Fos/Jun) functions in inflammatory bone and skin disease. Arthritis Res. Ther..

[15] Papoudou-Bai A., Hatzimichael E., Barbouti A., Kanavaros P. (2016). Expression patterns of the activator protein-1 (AP-1) family members in lymphoid neoplasms. Clin. Exp. Med..

[16] Moon Y.-M., Lee S.-Y., Kwok S.-K., Lee S.H., Kim D., Kim W.K., Her Y.-M., Son H.-J., Kim E.-K., Ryu J.-G. (2017). The Fos-Related Antigen 1–JUNB/Activator Protein 1 Transcription Complex, a Downstream Target of Signal Transducer and Activator of Transcription 3, Induces T Helper 17 Differentiation and Promotes Experimental Autoimmune Arthritis. Front. Immunol..

[17] Behmoaras J., Bhangal G., Smith J., McDonald K., Mutch B., Lai P.C., Domin J., Game L., Salama A., Foxwell B.M. (2008). Jund is a determinant of macrophage activation and is associated with glomerulonephritis susceptibility. Nat. Genet..

[18] Brown P.H., Kim S.H., Wise S.C., Sabichi A.L., Birrer M.J. (1996). Dominant-negative mutants of cJun inhibit AP-1 activity through multiple mechanisms and with different potencies. Cell Growth Differ. Mol. Boil. J. Am. Assoc. Cancer Res..

[19] Knapp J.I., Heppner C., Hickman A.B., Burns A.L., Chandrasekharappa S.C., Collins F.S., Marx S.J., Spiegel A.M., Agarwal S.K. (2000). Identification and characterization of JunD missense mutants that lack menin binding. Oncogene.

[20] Knebel B., Kotzka J., Knebel B., Hartwig S., Avci H., Jacob S., Nitzgen U., Schiller M., März W., Hoffmann M.M. (2013). A mutation in the c-Fos gene associated with congenital generalized lipodystrophy. Orphanet J. Rare Dis..

[21] Huber R., Kirsten H., Näkki A., Pohlers D., Thude H., Eidner T., Heinig M., Brand K., Ahnert P., Kinne R.W. (2019). Association of Human FOS Promoter Variants with the Occurrence of Knee-Osteoarthritis in a Case Control Association Study. Int. J. Mol. Sci..

[22] Kim K., Bang S.-Y., Lee H.-S., Bae S.-Y.B.H.-S.L.S.-C. (2017). Update on the genetic architecture of rheumatoid arthritis. Nat. Rev. Rheumatol..

[23] Busch R., Kollnberger S., Mellins E.D. (2019). HLA associations in inflammatory arthritis: emerging mechanisms and clinical implications. Nat. Rev. Rheumatol..

[24] Database of Single Nucleotide Polymorphisms (dbSNP). https://www.ncbi.nlm.nih.gov/snp/.

[25] Karczewski K.J., Francioli L.C., MacArthur D.G. (2020). The mutational constraint spectrum quantified from variation in 141,456 humans. Yearb. Paediatr. Endocrinol..

[26] The Genome Aggregation Database. https://gnomad.broadinstitute.org/.

[27] Hunt R.C., Simhadri V.L., Iandoli M., Sauna Z.E., Kimchi-Sarfaty C. (2014). Exposing synonymous mutations. Trends Genet..

[28] Supek F. (2015). The Code of Silence: Widespread Associations Between Synonymous Codon Biases and Gene Function. J. Mol. Evol..

[29] Quax T.E., Claassens N.J., Söll D., Van Der Oost J. (2015). Codon Bias as a Means to Fine-Tune Gene Expression. Mol. Cell.

[30] Gingold H., Pilpel Y. (2011). Determinants of translation efficiency and accuracy. Mol. Syst. Biol..

[31] Kunisch E., Pohlers D., Dunger S., Huber R., Kreusch A., Wiederanders B., Kinne R.W. (2002). What can experimental research offer to rheumatology today—The viewpoint of molecular biology? Contribution of molecular biology to pathogenesis research in rheumatology using the example of rheumatoid arthritis. Z. Rheumatol..

[32] Chung S.A., Shum A.K. (2016). Rare variants, autoimmune disease, and arthritis. Curr. Opin. Rheumatol..

[33] Kinne R.W., Liehr T., Beensen V., Kunisch E., Zimmermann T., Holland H., Pfeiffer R., Stahl H.-D., Lungershausen W., Hein G. (2001). Mosaic chromosomal aberrations in synovial fibroblasts of patients with rheumatoid arthritis, osteoarthritis, and other inflammatory joint diseases. Arthritis Res..

[34] Kinne R.W., Kunisch E., Beensen V., Zimmermann T., Emmrich F., Petrow P., Lungershausen W., Hein G., Braun R.K., Foerster M. (2003). Synovial fibroblasts and synovial macrophages from patients with rheumatoid arthritis and other inflammatory joint diseases show chromosomal aberrations. Genes Chromosom. Cancer.

[35] May G.H., Allen K.E., Clark W., Funk M., Gillespie D.A. (1998). Analysis of the interaction between c-Jun and c-Jun N-terminal kinase in vivo. J. Biol. Chem..

[36] Reva B., Antipin Y., Sander C. (2011). Predicting the functional impact of protein mutations: Application to cancer genomics. Nucleic Acids Res..

[37] (2001). Characteristics of amino acids. Curr. Protoc. Protein. Sci..

[38] Schauer M., Kottek T., Schönherr M., Bhattacharya A., Ibrahim S.M., Hirose M., Köhling R., Fuellen G., Schmitz U., Kunz M. (2015). A mutation in the NADH-dehydrogenase subunit 2 suppresses fibroblast aging. Oncotarget.

[39] Ginter T., Fahrer J., Kröhnert U., Fetz V., Garrone A., Stauber R.H., Reichardt W., Müller-Newen G., Kosan C., Heinzel T. (2014). Arginine residues within the DNA binding domain of STAT3 promote intracellular shuttling and phosphorylation of STAT3. Cell. Signal..

[40] Bossis G., Malnou C.E., Farras R., Andermarcher E., Hipskind R., Rodriguez M., Schmidt D., Muller S., Jariel-Encontre I., Piechaczyk M. (2005). Down-Regulation of c-Fos/c-Jun AP-1 Dimer Activity by Sumoylation. Mol. Cell. Biol..

[41] Ryseck R.P., Bravo R. (1991). c-JUN, JUN B, and JUN D differ in their binding affinities to AP-1 and CRE consensus sequences: Effect of FOS proteins. Oncogene.

[42] Eferl R., Wagner E.F. (2003). AP-1: A double-edged sword in tumorigenesis. Nat. Rev. Cancer.

[43] Bejjani F., Evanno E., Zibara K., Piechaczyk M., Jariel-Encontre I. (2019). The AP-1 transcriptional complex: Local switch or remote command?. Biochim. Biophys. Acta.

[44] Halazonetis T.D., Georgopoulos K., Greenberg M.E., Leder P. (1988). c-Jun dimerizes with itself and with c-Fos, forming complexes of different DNA binding affinities. Cell.

[45] Malemud C.J. (2018). The role of the JAK/STAT signal pathway in rheumatoid arthritis. Ther. Adv. Musculoskelet. Dis..

[46] Malemud C.J. (2013). Intracellular Signaling Pathways in Rheumatoid Arthritis. J. Clin. Cell. Immunol..

[47] Fang Q., Zhou C., Nandakumar K.S. (2020). Molecular and Cellular Pathways Contributing to Joint Damage in Rheumatoid Arthritis. Mediat. Inflamm..

[48] Aud D., Peng S.L. (2006). Mechanisms of Disease: Transcription factors in inflammatory arthritis. Nat. Clin. Pr. Rheumatol..

[49] Alonso I.G.D.L.F., Liang H.-C., Turner S.D., Lagger S., Merkel O., Kenner L. (2018). The Role of Activator Protein-1 (AP-1) Family Members in CD30-Positive Lymphomas. Cancers.

[50] Hamaratoglu F., Atkins M. (2020). Rounding up the Usual Suspects: Assessing Yorkie, AP-1, and Stat Coactivation in Tumorigenesis. Int. J. Mol. Sci..

[51] Giuliani C., Bucci I., Napolitano G., Giuliani C., Bucci I., Napolitano G. (2018). The Role of the Transcription Factor Nuclear Factor-kappa B in Thyroid Autoimmunity and Cancer. Front. Endocrinol..

[52] Huber R., Pietsch D., Panterodt T., Brand K. (2012). Regulation of C/EBPbeta and resulting functions in cells of the monocytic lineage. Cell Signal..

[53] Wei W., Jin J., Schlisio S., Harper J.W., Kaelin W.G. (2005). The v-Jun point mutation allows c-Jun to escape GSK3-dependent recognition and destruction by the Fbw7 ubiquitin ligase. Cancer Cell.

[54] Huber R., Stuhlmüller B., Kunisch E., Kinne R.W. (2020). Discrepancy between Jun/Fos Proto-Oncogene mRNA and Protein Expression in the Rheumatoid Arthritis Synovial Membrane. J.

[55] Firestein G.S., Echeverri F., Yeo M., Zvaifler N.J., Green D.R. (1997). Somatic mutations in the p53 tumor suppressor gene in rheumatoid arthritis synovium. Proc. Natl. Acad. Sci. USA.

[56] Han Z., Boyle D.L., Shi Y., Green D.R., Firestein G.S. (1999). Dominant-negative p53 mutations in rheumatoid arthritis. Arthritis Rheum..

[57] Yamanishi Y., Boyle D.L., Rosengren S., Green D.R., Zvaifler N.J., Firestein G.S. (2002). Regional analysis of p53 mutations in rheumatoid arthritis synovium. Proc. Natl. Acad. Sci. USA.

[58] McGonagle D., Watad A., Savic S. (2018). Mechanistic immunological based classification of rheumatoid arthritis. Autoimmun. Rev..

[59] Uspenskaya N.Y., Akopov S.B., Snezhkov E.V., Sverdlov E.D. (2019). The Rate of Human Germline Mutations—Variable Factor of Evolution and Diseases. Russ. J. Genet..

[60] Ferguson L.R. (2010). Chronic inflammation and mutagenesis. Mutat. Res. Mol. Mech. Mutagen..

[61] Carlson J., Locke A.E., Flickinger M., Zawistowski M., Levy S., Myers R.M., Boehnke M., Kang H.M., Scott L.J., Jun Z.L. (2018). Extremely rare variants reveal patterns of germline mutation rate heterogeneity in humans. Nat. Commun..

[62] Huber R., Hummert C., Gausmann U., Pohlers D., Koczan D., Guthke R., Kinne R.W. (2008). Identification of intra-group, inter-individual, and gene-specific variances in mRNA expression profiles in the rheumatoid arthritis synovial membrane. Arthritis Res. Ther..

[63] Mankia K., Emery P. (2016). Review: Preclinical Rheumatoid Arthritis: Progress Toward Prevention. Arthritis Rheumatol..

[64] Zimmermann T., Kunisch E., Pfeiffer R., Hirth A., Stahl H.-D., Sack U., Laube A., Liesaus E., Roth A., Palombo-Kinne E. (2000). Isolation and characterization of rheumatoid arthritis synovial fibroblasts from primary culture—Primary culture cells markedly differ from fourth-passage cells. Arthritis Res..

[65] Arnett F.C., Edworthy S.M., Bloch D.A., McShane D.J., Fries J.F., Cooper N.S., Healey L.A., Kaplan S.R., Liang M.H., Luthra H.S. (1988). The american rheumatism association 1987 revised criteria for the classification of rheumatoid arthritis. Arthritis Rheum..

[66] Altman R., Asch E., Bloch D., Bole G., Borenstein D., Brandt K., Christy W., Cooke T.D., Greenwald R., Hochberg M. (1986). Development of criteria for the classification and reporting of osteoarthritis: Classification of osteoarthritis of the knee. Arthritis Rheum..

[67] Huber R., Kunisch E., Gluck B., Egerer R., Sickinger S., Kinne R.W. (2003). Comparison of conventional and real-time RT-PCR for the quantitation of jun protooncogene mRNA and analysis of junB mRNA expression in synovial membranes and isolated synovial fibroblasts from rheumatoid arthritis patients. Z. Rheumatol..

[68] Haas S.C., Huber R., Gutsch R., Kandemir J.D., Cappello C., Krauter J., Duyster J., Ganser A., Brand K. (2010). ITD- and FL-induced FLT3 signal transduction leads to increased C/EBPbeta-LIP expression and LIP/LAP ratio by different signalling modules. Br. J. Haematol..

[69] Umesono K., Murakami K.K., Thompson C.C., Evans R.M. (1991). Direct repeats as selective response elements for the thyroid hormone, retinoic acid, and vitamin D3 receptors. Cell.

[70] De Martin R., Strasswimmer J., Philipson L. (1993). A new luciferase promoter insertion vector for the analysis of weak transcriptional activities. Gene.

[71] White L.A., E Brinckerhoff C. (1995). Two activator protein-1 elements in the matrix metalloproteinase-1 promoter have different effects on transcription and bind Jun D, c-Fos, and Fra-2. Matrix Biol..

[72] Dungera S., Neumannb S., Zellc R., Hirschfeldc E.B.-, Stelznerc A., Paschkeb R., Kinne R.W., Sickingera S. (2001). Mutation Detection in Mosaic Situations: RNA Mismatch Assay and Denaturing Gradient Gel Electrophoresis Are More Sensitive Than Conventional Cycle Sequencing. Anal. Biochem..

[73] Poxon S.W., Hughes J.A. (1999). A biofunctional assay to study pRL-CMV plasmid DNA formulation stability. PDA. J. Pharm. Sci. Technol..

[74] Kunisch E., Jansen A., Kojima F., Löffler I., Kapoor M., Kawai S., Rubio I., Crofford L.J., Kinne R.W. (2009). Prostaglandin E2 Differentially Modulates Proinflammatory/Prodestructive Effects of TNF-α on Synovial Fibroblasts via Specific E Prostanoid Receptors/cAMP. J. Immunol..

[75] Haas C., Aicher W.K., Dinkel A., Eibel H., Peter H.H. (1997). Characterization of SV40 T antigen immortalized human synovial fibroblasts: Maintained expression patterns of EGR-i, HLA-DR and some surface receptors. Rheumatol. Int..

[76] Pohlers D., Schmidt-Weber C.B., Franch A., Kuhlmann J., Brauer R., Emmrich F., Kinne R.W. (2002). Differential clinical efficacy of anti-CD4 monoclonal antibodies in rat adjuvant arthritis is paralleled by differential influence on NF-kappaB binding activity and TNF-alpha secretion of T cells. Arthritis Res..

[77] Huber R., Panterodt T., Welz B., Christmann M., Friesenhagen J., Westphal A., Pietsch D., Brand K. (2015). C/EBPbeta-LAP*/LAP Expression Is Mediated by RSK/eIF4B-Dependent Signalling and Boosted by Increased Protein Stability in Models of Monocytic Differentiation. PLoS ONE.

[78] Kirsten H., Dienst S., Emmrich F., Ahnert P. (2006). CalcDalton: A tool for multiplex genotyping primer design for single-base extension reactions using cleavable primers. Biotechniques.

[79] Kirsten H., Teupser D., Weissfuss J., Wolfram G., Emmrich F., Ahnert P. (2006). Robustness of single-base extension against mismatches at the site of primer attachment in a clinical assay. J. Mol. Med..

