# Supplementary material for: Identification of New, Functionally Relevant Mutations in the Coding Regions of the Human Fos and Jun Proto-Oncogenes in Rheumatoid Arthritis Synovial Tissue
Source: Life (Basel). 2020 Dec 23;11(1):5. doi: 10.3390/life11010005 (PMC7823737; doi:10.3390/life11010005)
Supplement: Supplementary file 1 [file life-11-00005-s001.pdf]

Table S1. Clinical Characteristics of the Donors.

		normal controls (NC)	osteoarthritis (OA)	rheumatoid arthritis (RA)
**donors (n)**		484	288	298
gender (female/male)		226/258	204/84	216/82
age (years ± SEM)		41.2 ± 0.5	69.1 ± 0.5	59.7 ± 0.7
disease duration (years ± SEM)		-	5.9 ± 0.4 (n.d.: 5)	11.5 ± 0.6 (n.d.: 2)
rheumatoid factor (positive/negative/n.d.)		0/0/484	21/267/0	219/54/25
ESR[1] (mm/h ± SEM)		n.d.	16.4 ± 0.8	26.4 ± 1.2 (n.d.: 4)
CRP[2] (mg/l ± SEM)		n.d.	7.6 ± 1.3	17.3 ± 1.4 (n.d.: 17)
ARA[3] - criteria for RA (n ± SEM)		n.d.	0.1 ± 0.01	4.7 ± 0.1 (n.d.: 2)
Concomitant medication[4]	MTX[5] (n)	-	0	193
	Steroids	-	2	183
	NSAIDs[6] (n)	-	141	230

n.d. not determined

[1] Erythrocyte sedimentation rate.

[2] C-reactive protein, normal range: < 5 mg/l.

[3] American Rheumatism Association (now: American College of Rheumatology).

[4] n.d.: 8 (RA).

[5] Methotrexate.

[6] non-steroidal anti-inflammatory drugs.

TableS2. Primer Sequences and Specific PCR Conditions for NIRCA and Functional Analyses.

gene	primer forward (5′→3′)	primer reverse (3′→5′)	product (base pairs)	amplification protocol (45 cycles)
cjun	5′- CGTGAAGTGAC-GGACTGTTC - 3′	5′- CTTCAAAATGTTTGCAACTG -3′	1020	denaturation: 45 s, 95°C, primer annealing: 45 s, 52°C, amplification: 180 s, 72°C
junB	5′- ATGTGCAC-TAAAATGGAACAG -3′	5′- CAGGGGAC-GTTCAGAAGGCGTGT -3′	1054	denaturation: 45 s, 95°C, primer annealing: 45 s, 52°C, amplification: 180 s, 72°C
junD	5′- ATGGAAACACCCTTC-TACGGC -3′	5′- CGGACTCAGTACGCGGG-CACCTG -3′	1049	denaturation: 45 s, 95°C, primer annealing: 45 s, 60°C, amplification: 180 s, 72°C
cfos	5′-ATGATGTTCTCGGGCTTCAA -3′	5′- TCACAGGGCCAG-CAGCGGGT -3′	1140	denaturation: 45 s, 95°C, primer annealing: 45 s, 52°C, amplification: 180 s, 72°C

For NIRCA analyses, sequences were cloned into the vector pUC 19 (primer forward: binding site for T7 RNA polymerase + *Eco* RI restriction site + sequence for primer binding; primer reverse: binding site for T7 RNA polymerase + *Bam* HI restriction + sequence for primer binding).
For functional analyses, sequences were cloned into the vector pUBT-luc (primer forward: Hind III restriction site + sequence for primer binding; primer reverse: Not I restriction + sequence for primer binding).

Table 3. Primer Sequences and Specific Conditions for Genotyping.

gene	mutation	PCR primer forward (5′→3′)	PCR primer reverse (3′→5′)	Genotyping Primer
FOS	fos73	5′-ACGTT-GGATGGCCAACTTCATTCCCAC -3′	5′- ACGTT-GGATGACTCCGAAAGGGTGAGG -3′	5′- bioGGCT[L]CAC-CAGCCACTGCA -3′
FOS	fos125	5′-ACGTTGGATGAAGAC-CATGACAGGAGGC -3′	5′- ACGTTGGATGTCATCCTCTG-TACTGGGC -3′	5′- bioCCTGTT[L]CACCTT-GCCCCTC -3′

(Bio, biotin; [L], photo cleavable linker; PCR product size: fos73 - 142 base pairs, fos125 – 165 base pairs).
